# The effectiveness of virtual reality–assisted relaxation therapy in improving psychological symptoms and fatigue of breast cancer survivors: a systematic review and meta-analysis

**DOI:** 10.3389/fonc.2026.1927287

**Published:** 2026-10-23

**Authors:** Yu Deng, Ying Chen, Jiangling Mo, Yinyin Liang, Sijie Yu, Xue Bai

**Affiliations:** The Second Affiliated Hospital of Chongqing Medical University, Chongqing, China

**Keywords:** breast cancer, meta-analysis, psychological symptoms, relaxation therapy, VR

## Abstract

**Objective:**

This meta-analysis investigated the effect of virtual reality–assisted relaxation therapy (VR-ART) on the reduction of psychological symptoms and fatigue in breast cancer survivors.

**Methods:**

This review followed PRISMA 2020 reporting guidelines. We searched 9 bibliographic databases, plus ClinicalTrials.gov, WHO ICTRP, and GreyGuide, for records published from database inception through September 2026, limited to Chinese- and English-language publications. Backward and forward citation chasing of included studies and related reviews was completed via Web of Science to identify additional eligible records. Parallel-group RCTs evaluating VR-assisted relaxation therapy on psychological symptoms (anxiety, depression, psychological distress) and cancer-related fatigue among breast cancer survivors were included. Two reviewers independently assessed trial risk of bias using the Cochrane RoB 2 tool.

**Results:**

Ten RCTs with 995 breast cancer survivors were included. Pooled estimates showed VR-ART yielded statistically significant reductions in anxiety (SMD = −1.94, 95% CI [−2.49, −1.40]; *P* < 0.001), depressive symptoms (SMD = −1.11, 95% CI [−1.36, −0.87]; *P* < 0.001), and psychological distress (MD = −1.33, 95% CI [−1.99, −0.67]; *P* = 0.005) versus controls. effect sizes varied across treatment stages; heterogeneity for anxiety and psychological distress disappeared among trials in active treatment stages; heterogeneity for anxiety and psychological distress disappeared among trials in active treatment stages (anxiety: *P* = 0.91, I² = 0%; psychological distress: *P* = 0.79, I² = 0%). Combining sensitivity analysis and existing research, we hypothesized that the combination of chemotherapy regimen and VR interactivity might be associated with variability in effect sizes for psychological distress (P = 0.96, I² = 0%); this observation was exploratory and hypothesis-generating only. Fatigue was synthesized qualitatively rather than pooled quantitatively due to high heterogeneity, with studies reporting reduced self-reported fatigue.

**Conclusion:**

VR-ART was associated with statistically significant reductions in anxiety, depression, psychological distress, and cancer-related fatigue among breast cancer survivors. Based on findings from exploratory subgroup analyses, we speculated that intervention timing, VR interactivity, and concurrent chemotherapy status might influence the magnitude of intervention effects. Formal testing remains absent, and further research is required to develop individualized intervention strategies and optimize treatment responses.

**Systematic review registration:**

https://www.crd.york.ac.uk/PROSPERO/, identifier CRD420251114925.

## Introduction

Out of nearly 20 million new cancer cases worldwide in 2022 (1), approximately 2.308 million were new cases of breast cancer in women, accounting for 11.6% of all new cancer diagnoses globally (2). With advances in cancer treatment technology and an aging population, the incidence of cancer-related mental health issues is expected to continue rising in the coming years (3). Breast cancer survivors (BCSs) were broadly defined as individuals with a confirmed diagnosis of breast cancer from the time of diagnosis to the end of life, including patients undergoing active anti-cancer treatment, those who had finished primary treatment and were in follow-up, and individuals living with metastatic breast cancer (4). As the disease progresses, BCSs experience declines in physical and mental function and quality of life (5). However, the current model of care for individuals with breast cancer primarily emphasizes disease treatment and subsequent ongoing surveillance to detect recurrence, paying insufficient attention to patients’ psychological and functional well-being (6). The cancer experience and physical disability lead to the low self-esteem (7, 8). A substantial proportion of BCSs experience persistent cancer-related symptom burden, including physical symptoms (pain, fatigue) and psychological symptoms (elevated anxiety and depressive symptoms) (9). These symptoms emerge from the complex interplay of biological, psychological, and social factors across the survivorship trajectory. A growing body of evidence indicates that persistent psychological distress and co-occurring cancer-related fatigue are consistently associated with impaired health-related quality of life among BCSs (10, 11). Similarly, Studies have confirmed that the psychological symptoms and fatigue of cancer patients increase their suicide risk by 4.4 times compared to the general population (12). The anxiety and depression risks of BCSs significantly increase within 2 and 4 years after diagnosis (13). It is particularly urgent to explore intervention measures that can effectively alleviate the psychological symptoms and fatigue of BCSs.

Currently, antidepressants are only modestly effective for some patients (14), and the long-term benefits of cognitive behavioral therapy are limited (15). Therefore, a single psychological intervention is often insufficient to address the emotional management needs of BCSs. Virtual reality (VR) technology has been shown to benefit the long-term rehabilitation of BCSs (16). VR use computers to create virtual environments, enabling users to interact virtually through visual, auditory, and tactile simulations. Currently, this technology has been combined with psychological interventions and is being applied in medical fields, such as preoperative simulation, relaxation therapy, and game training and has demonstrated effective results (17–19). Obviously, the virtual reality–assisted relaxation therapy (VR-ART) is commonly used. Among psychological interventions, relaxation therapy aims to improve the psychological state and promote recovery by guiding patients to relax their bodies and minds (20). Its principle lies in enhancing the excitability of the parasympathetic nerve, causing muscle relaxation and balancing neuroendocrine regulation. Felix et al. reported that relaxation therapy could effectively alleviate pain-related negative emotions (21). Furthermore, Fabi observed that patients equipped with VR goggles who received relaxation guidance and focused on pleasant content experienced reduced negative perceptions (22). This intervention was associated with a low risk of adverse events, and prior evidence supported its safety profile (23). These factors contributed to its growing adoption in the clinical care of BCSs.

Existing evidence supports the efficacy of VR therapy in reducing anxiety and depression among BCSs and improving their quality of life. Nevertheless, the included interventions in previous studies were diverse, resulting in substantial heterogeneity. Accordingly, this meta-analysis narrows the scope to VR-ART to assess its impact on negative emotions in BCSs, thereby guiding future research and confirming the safety and effectiveness of VR-assisted interventions.

## Methods

### Protocol and guidance

This meta-analysis was conducted according to the Preferred Reporting Items for Systematic Reviews and Meta-Analyses (PRISMA) reporting guidelines. The review protocol was registered on PROSPERO (CRD420251114925), with Version 1.0(11 August 2025). We added the keyword “Relaxation Therapy” in Version 1.1(17 November 2025). The registered protocol contained relatively broad descriptions of participants, interventions and study settings. Prior to commencing literature screening, the review team refined and clarified these broad items to standardize study selection judgements, without modifying the core pre-specified eligibility framework. Specifically, the tentative 70-year upper age limit was removed to include all adult BCSs aged 18 years or older, improving the generalizability of the review findings. The originally broad definition of VR interventions was refined to focus on VR-assisted relaxation therapy, while other forms of VR interventions were excluded. We also clarified the study setting to exclude studies performed under COVID-19 lockdowns where intervention delivery departed from routine clinical environments. These clarifications have now been documented in PROSPERO Version 2.0 (28 September 2026). The full search was re-executed on 26 September 2026, and the updated search results were adopted for this systematic review and meta-analysis.

### Ethics statement

Ethical approval was not required for this study as it is based on published data from previously approved studies and does not involve any direct interaction with human participants.

### Data sources and search strategies

We conducted an updated search of nine electronic databases in September 2026, including PubMed, Web of Science, EMBASE, CINAHL, Cochrane Library, China National Knowledge Infrastructure (CNKI), Wanfang Data, China Science and Technology Journal Database (VIP), and SinoMed, for records published from database inception. We also conducted supplementary searches on ClinicalTrials.gov, the WHO ICTRP clinical trial registration platform, and GreyGuide for grey literature. This search focused on evaluating the effectiveness of VR-ART in enhancing the emotional well-being of patients with breast cancer. We conducted the search by combining MeSH terms and free-text keywords. At the same time, a retrospective search was conducted on the references and related reviews included in the study, and the cited literature was obtained through Web of Science. The filter is restricted to articles in Chinese and English to minimize bias introduced during translation, with primary search terms including breast cancer, virtual reality technology, VR, virtual environment, psychological rehabilitation, relaxation therapy, negative emotions, affective disorders, intervention measures, and randomized controlled trials. The full strategies for the databases are listed in **Supplementary Materials**.

### Study selection

A total of 1, 360 studies were retrieved from the databases, The search retrieved 41 records from ClinicalTrials.gov and 52 records from the WHO ICTRP. No relevant records were identified from GreyGuide. Manual screening of the reference lists of eligible studies yielded an additional 3 records. Two researchers independently conducted literature screening, data extraction, and risk-of-bias assessment. Discrepancies were first resolved through discussion; persistent disagreements were adjudicated by a third independent reviewer. The full-text articles of potentially relevant studies were subsequently reviewed. Disagreements were resolved through discussion or consultation with additional researchers. Finally, a quality assessment of the included studies was performed.

### Inclusion and exclusion criteria

Based on the research topic, two investigators developed the inclusion and exclusion criteria for the literature review. VR-ART was operationally defined as a relaxation-focused intervention delivered via virtual reality hardware, with immersive scene presentation, head-mounted display devices, and passive or low-interaction relaxation content as core components. The Patient, Intervention, Control, Outcome, and Study Design (PICOS) framework was used to guide the formulation of inclusion criteria: (P) patients with confirmed breast cancer from diagnosis to death, including those receiving active anti-cancer treatment, survivors post-primary treatment under follow-up, and individuals with metastatic disease, aged ≥18 years; (I) experimental group receiving VR combined with any type of relaxation therapy intervention; (C) control group receiving standard care or no intervention; (O) primary outcomes including negative emotion-related indicators such as anxiety, depression, fatigue, and psychological distress; and (S) randomized controlled trials(RCTs). This framework helps researchers to find relevant evidence by breaking down complex clinical problems into structured, searchable components. The exclusion criteria were as follows (1): studies that did not clearly specify the type of cancer in the research subjects (2); studies in which the experimental group received only basic VR-based games or health education, without detailed intervention and control measures, or those that did not report mean values, standard deviations(SD), median values, quartiles, range, or 95% confidence intervals (3); simple VR exposure therapy, independent mindfulness training, simple music therapy, traditional rehabilitation training, and pure digital information intervention (4); study types such as conference abstracts, posters, reviews, letters, or study protocols.

We excluded studies conducted during the COVID-19 pandemic, as universal population-level emotional fluctuations and pandemic-related shifts in clinical care models during that period could have confounded outcome measures and biased VR-ART effect estimates (24). This exclusion criterion was pre-specified in the original registered protocol.

### Data extraction

Two researchers independently extracted the change scores of mean values and SD using Excel spreadsheets. Data for each included trial were extracted at the immediate post-intervention time point to minimize confounding introduced by extended follow-up periods. For studies reporting only baseline and endpoint means and standard deviations, change scores were calculated using a standardized formula: mean change = mean(baseline) − mean(follow-up), SD= $\sqrt{SD{\left(B\right)}^{2}+SD{\left(F\right)}^{2}-(2\times R\times SD(B)\times SD(F))}$. We assumed a pre-post correlation of 0.5 when calculating change-score standard deviations, consistent with Cochrane Handbook recommendations for pre-post outcome data in cancer populations (25). Median values, quartiles, range, or 95% confidence intervals should also be converted into mean values and SD. Following standard conversion methods recommended in the Cochrane Handbook, for studies reporting only medians, total ranges, and interquartile ranges (IQRs), study medians were used as approximate estimates of study means. Standard deviations were estimated from reported IQRs as SD = (Q3 − Q1)/1.35, and from reported total ranges as SD = (maximum − minimum)/4 (25). These data included research characteristics (first author, publication date, and research location), demographic characteristics (sample size, age, and treatment phase), and intervention details (VR content, intervention duration, and measurement tools). The primary outcomes included the change scores of the anxiety, depression, fatigue, and psychological distress evaluated using commonly clinical scales. In the event of missing values in the included literature, researchers should contact the original authors to acquire the complete data. Should the data remain unobtainable, the corresponding literature must be excluded from the analysis. In cases of disagreement, a third party was consulted and the original literature was referenced for verification. Raw extracted data and transformed statistical values were presented in **Supplementary Table 1**.

### Risk of bias assessment

We used the Cochrane Risk of Bias 2 (RoB 2) tool to evaluate the methodological quality of included studies, comprising five domains (1): randomisation process (2), deviations from intended interventions (3), missing outcome data, (4) measurement of the outcome, (5) selection of the reported result. Each entry is evaluated as “low risk, “ “some concerns, “ or “high risk”. Based on the assessment in five domains, the researchers determined the final overall risk of bias. “Fully low risk” is classified as low risk, “No high risks, but at least one concern” is classified as some concerns, and “At least one high-risk factor, or multiple concerns” was classified as high risk. The assessments were performed by two independent reviewers.

### GRADE assessment

Two reviewers independently rated the certainty of evidence for all prespecified outcomes using the GRADE framework. Discrepancies in ratings were resolved via discussion, with arbitration by a third reviewer if consensus could not be reached. Evidence certainty was categorized into four ordinal levels (high, moderate, low, very low), with downgrading applied across five standard GRADE domains: risk of bias, inconsistency, indirectness, imprecision, and risk of publication bias.

### Statistical analysis

We used Review Manager 5.4 provided by the Cochrane Collaboration for effect analysis and heterogeneity testing. Between-group differences were expressed as standardized mean differences (SMD) or mean differences (MD) with 95% confidence intervals (CI) as effect measures. Simultaneously, Q-tests and I² values were used to assess the heterogeneity of the effect sizes. Following Cochrane Handbook recommendations, when *P* ≤ 0.1 and I² > 50%, this indicates substantial heterogeneity among studies, warranting the use of a random-effects model. To minimize the interference caused by differences among the intervention schemes, this study employed the random effects model. Subgroup and sensitivity analyses were performed. Subgroup analyses were purely exploratory and hypothesis-generating, with no formal tests for subgroup differences. Sensitivity analyses were conducted to assess the robustness of pooled estimates. Considering that the improvement effects of negative emotions may be influenced by sociocultural background, timing of intervention, and VR content, we conducted subgroup analyses stratified by these factors. If subgroup analysis cannot be performed, qualitative analysis should be used instead of meta-analysis. As each indicator included fewer studies, publication bias was assessed quantitatively. While conducting the qualitative analysis using the funnel plot, we used Stata 18.0, to conduct Egger’s test to evaluate publication bias. The results of the meta-analysis are presented as forest plots. Statistical significance was determined as a 2-sided *p*-value of < 0.05.

## Results

### Articles’ screening results

After removing duplicates using EndNote 21.0, 942 articles remained. Following a review of the titles and abstracts, we excluded studies irrelevant to the topic and non-RCT research, resulting in 53 articles. After reading the full text, we rigorously screened studies according to the inclusion criteria. After adding 3 eligible studies identified through manual citation chaining, a total of 10 studies were ultimately included in the final synthesis: 2 published in Chinese and 8 in English. Full abstracts and accessible source links for the two Chinese-language studies were provided in the **Supplementary Materials**. The complete study screening workflow was illustrated in the PRISMA flow diagram (**Figure 1**).

**Figure 1:**
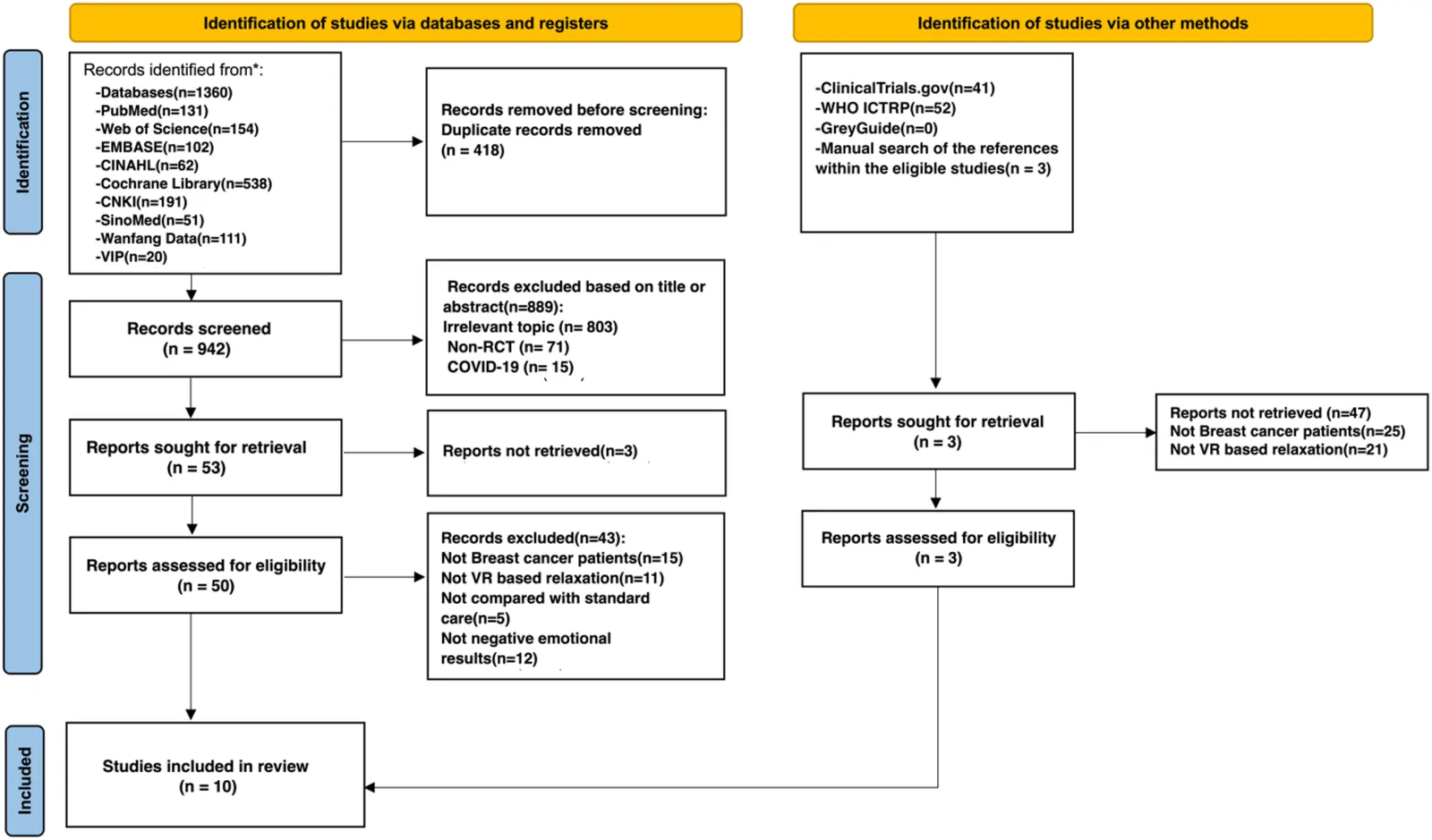
Flow diagram of the studies’ selection process.

### Studies characteristics and quality assessment

This study included 10 RCTs (26–35), involving 995 patients. Among these, six studies (26, 27, 30–32, 35) applied interventions at any stage of treatment, while four (28, 29, 33, 34) restricted interventions to the initial treatment phase. Owing to the unique nature of psychological intervention methods, three studies (31, 34, 35) did not employ blinding, while the remaining seven (26–30, 32, 33) utilized single-blind designs. Except for two studies (32, 33) that showed significant differences in baseline age and treatment duration, no significant differences were observed in the baseline characteristics. All the control groups received standard care. The average age of the participants ranged from 43.6 to 72.9 years. The primary VR devices used were headsets and VR goggles paired with handheld controllers. The average duration of each intervention ranged from 22.2 to 25.3 minutes, with intervention periods varying from 2 weeks to 3 months. The follow-up period lasted up to six months after the intervention. The basic characteristics of this study were listed in **Table 1**.

**Table 1:** Characteristics of the included studies.

Study	Area	Stage	n, M/F	Accepted treatment	Treatment time	Follow-up	Groups	Case	Age	Main outcomes	Types of relaxation	Intervention
Uslu, 2023 (26)	Turkey	Any	66, 0/66	CA protocol in chemotherapy	4 cycles	NR	VR	33	53.42 (9.25)	STAI, CFS	Natural environment relaxation training	Weekly group sessions, lasting 30 minutes each time
							SC	33	51.42 (8.53)			
Czech, 2024 (27)	Poland	Any	33, 0/33	Any	2 weeks	8 weeks	VR	17	56.68 (11.26)	HADS-M	Natural environment relaxation training	A total of 8 sessions, each lasting 15 minutes
							SC	16	59.88 (10.72)			
Li Mengdan, 2024 (28)	China	First Chemotherapy	246, 0/246	Chemotherapy	3 months	6 months	VR	121	53.6 (9.4)	DT	Natural environment relaxation training and information guidance	Once or twice a week, for 15–20 minutes per session (at least 12 times before T1)
							SC	125	55.2 (9.7)			
Torres, 2024 (29)	Spain	First Chemotherapy	133, 0/133	Chemotherapy	4 cycles	NR	VR	71	49 (11.7)	HADS-M	Natural environment relaxation training and information guidance	4 repetitions of intervention, each lasting 30 to 45 minutes
							SC	62	50 (11.6)			
Zhang Xiuqing, 2022 (30)	China	Any	77, 0/77	≥2 courses of Chemotherapy	3 months	NR	VR	38	52.29 (7.69)	DT, PFS, SAS, SDS	Natural environment relaxation training and information guidance	6 times, 30 minutes each time. (at least 3 times in the first month)
							SC	39	51.03 (7.98)			
Yu Lizhen, 2024 (31)	China	Any	60, 0/60	First unilateral mastectomy	2 months	NR	VR	30	47.29 (3.67	SAS, SDS	Natural environment relaxation training and mindfulness techniques	A total of 7 sessions, weekly group sessions, each 30 minutes
							SC	30	46.14 (4.09)			
Wu Shanshan, 2025 (32)	China	Any	85, 0/85	Chemotherapy	(5 ± 2) weeks	NR	VR	44	43.6 (11.1)	DT	Natural environment relaxation training	A total of 3 to 5 times, with each session lasting (15 ± 3) minutes
							SC	41	49.3 (9.4)			
Grynne, 2025 (33)	Sweden	First Radiotherapy	111, 0/111	Radiotherapy	2 weeks	6 months	VR	59	60.2 (10.7)	DT	VR exposure therapy and cognitive relaxation	Before and after each radiotherapy session, for 15–20 minutes per session
							SC	52	61.7 (9.18)			
Chirico, 2020 (34)	Italy	First Chemotherapy	62, 0/62	CA protocol in adjuvant chemotherapy	4 cycles	NR	VR	28	55.18 (5.70)	SAI	Natural environment relaxation training	Once every cycle, lasting for 20 minutes each time
							SC	34	56.2 (6.79)			
Fu Sijia, 2025 (35)	China	Any	122, 0/122	CMF protocol in chemotherapy	8 weeks	NR	VR	61	72.5 (6.20)	CFS	Natural environment relaxation training and mindfulness techniques	1–2 times per day, 25 minutes per session
							SC	61	72.9 (6.10)			

Chemotherapy acronyms: CA, cyclophosphamide plus doxorubicin; CMF, cyclophosphamide-methotrexate-flourouracil; Other acronyms: F, female; M, male; NR, not reported; STAI, State-Trait Anxiety Inventory scores; CFS, Cancer Fatigue Scale; PFS, Piper Fatigue Scale; HADS-M, Hospital Anxiety and Depression Scale; DT, Distress Thermometer; SAS, Self-Rating Anxiety Scale; SDS, Self-rating Depression Scale; SAI, Spielberger State Anxiety Inventory.

Two researchers conducted quality assessments using the ROB2 tool, with 3 studies rated as low risk of bias, and 7 studies rated as some concerns. None of the included studies implemented outcome assessor blinding for these subjective self-reported outcomes, introducing measurable detection bias risk; this was the primary driver of “some concerns” RoB 2 judgments across trials. The quality grades of the included studies were shown in **Supplementary Figure 1**.

### Anxiety

Six studies (26, 27, 29–31, 34) used anxiety scores as the outcome measures. Owing to differences in measurement tools, SMD was employed to analyze treatment efficacy. Overall, anxiety scores on the BCSs decreased significantly after treatment (SMD = -1.94; 95% CI [-2.49, -1.40]; *P* < 0.001) (**Figure 2**). Subgroup analyses were performed based on area and treatment phase. The results indicated that area had little effect on heterogeneity (**Table 2**). Subsequently, after conducting subgroup analyses by intervention phase, the any-treatment-phase group showed no significant heterogeneity (*P* = 0.91, I²= 0%) (**Supplementary Figure 2**). However, heterogeneity decreased but remained present (*P* = 0.11, I²= 61%) in the first-treatment-phase group. According to the analysis, the first treatment phase group included one three-arm RCT (34), which may cause some heterogeneity. The effect sizes for both the first-treatment-phase group (SMD = -2.80; 95% CI[-3.5, -2.09]; *P* < 0.01) and the any-treatment-phase group (SMD = -1.5; 95% CI[-1.79, -1.21]; *P* < 0.01) were statistically significant (**Table 3**).

**Figure 2:**
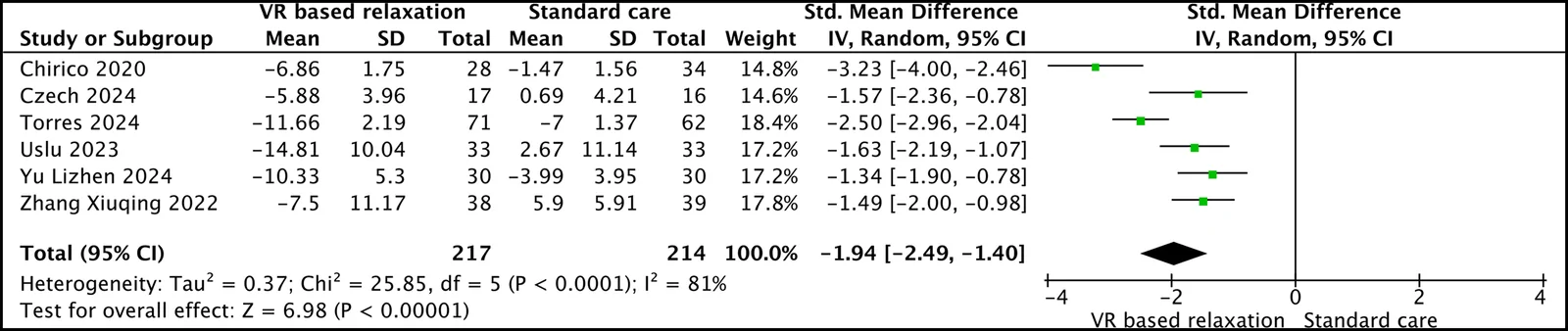
Meta-analysis of anxiety levels among BCSs: VR-relaxation versus standard care.

**Table 2:** Heterogeneity test of subgroup analyses.

Outcomes		No. of study	Heterogeneity test	
Subgroup			I^2^ (%)	P(H)
Anxiety				
	Overall	6	81	< 0.0001
Area	Asia	3	0	0.77
	Europe	3	77	0.01
Intervention stage	Initial treatment stage	2	61	0.11
	Any treatment stage	4	0	0.91
Depression				
	Overall	4	25	0.26
Area	Asia	2	25	0.25
	Europe	2	0	0.39
Intervention stage	Initial treatment stage	1	NA	NA
	Any treatment stage	3	44	0.17
Psychological distress				
	Overall	4	63	0.05
Area	Asia	3	71	0.03
	Europe	1	NA	NA
Intervention stage	Initial treatment stage	2	0	0.79
	Any treatment stage	2	41	0.19

NA, not available.

**Table 3:** Effect size of subgroup analyses.

Outcomes			SMD or MD (95%CI)	*P* value
Subgroup		No. of study		
Anxiety				
	Overall	6	-1.94 (-2.49, -1.40)	<0.00001
Area	Asia	3	-1.49 (-1.8, -1.17)	< 0.00001
	Europe	3	-2.44 (-3.24, -1.64)	< 0.00001
Intervention stage	Initial treatment stage	2	-2.8 (-3.5, -2.09)	< 0.00001
	Any treatment stage	4	-1.5 (-1.79, -1.21)	< 0.00001
Depression				
	Overall	4	-1.11 (-1.36, -0.87)	< 0.00001
Area	Asia	2	-0.94 (-1.35, -0.53)	< 0.00001
	Europe	2	-1.28 (-1.61, -0.94)	< 0.00001
Intervention stage	Initial treatment stage	1	-1.21 (-1.58, -0.84)	< 0.00001
	Any treatment stage	3	-1.09 (-1.54, -0.64)	< 0.00001
Psychological distress				
	Overall	4	-1.33 (-1.99, -0.67)	< 0.0001
Area	Asia	3	-1.45 (-2.30, -0.61)	0.0007
	Europe	1	-0.89 (-1.85, 0.07)	0.07
Intervention stage	Initial treatment stage	2	-1.01 (-1.46, -0.55)	< 0.0001
	Any treatment stage	2	-1.79 (-2.80, -0.79)	0.0005

CI, confidence interval; SMD, Standardized mean difference; MD, Mean Difference.

### Depression

Outcome measures related to depression were reported in four studies (27, 29–31). Despite modest between-study heterogeneity (*P* = 0.26, I²=25%), a random-effects model was used to pool all effect estimates, given clinical and methodological diversity across included trials. VR intervention was associated with a statistically significant reduction in depressive symptoms among BCSs (SMD = -1.12; 95% CI [-1.41, -0.83]; *P* < 0.001) (**Figure 3**). We conducted subgroup analyses by area for the improvement of depression. The Asian group (*P* = 0.25, I²=25%) (SMD= -0.94; 95% CI [-1.35, -0.53); *P* < 0.01)]and the European group (*P* = 0.39, I²= 0%) (SMD= -1.28; 95% CI [-1.61, -0.94]; *P* < 0.01) showed no significant heterogeneity, with statistically significant improvement in depression in both groups. (**Table 3**).

**Figure 3:**
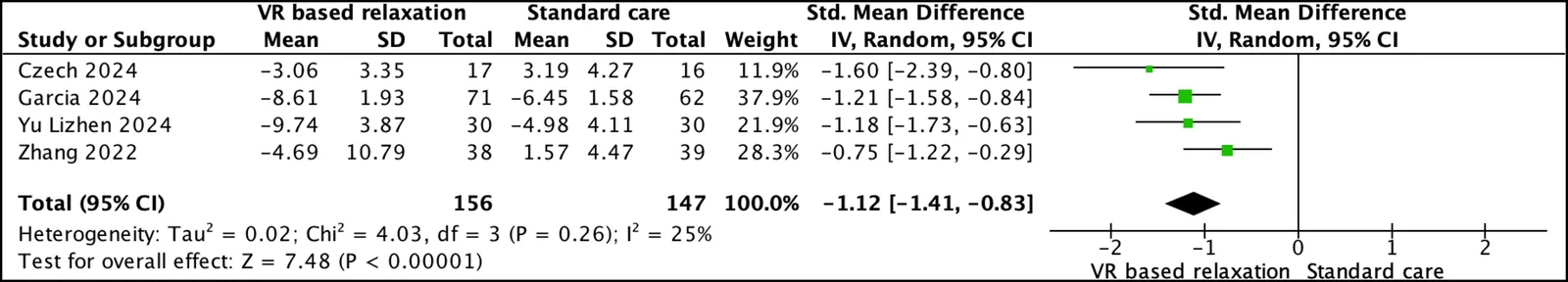
Meta-analysis of depression levels among BCSs.

### Psychological distress

Four studies (28, 30, 32, 33) were included regarding psychological distress levels. Because all studies employed the Distress Thermometer (DT) as the measurement tool, we analyzed the differences in changes using the MD. Due to the high heterogeneity (*P* = 0.05, I²= 63%), a random-effects model was used for data analysis. The results showed a significant reduction in psychological distress (MD= -1.33; 95% CI [-1.99, -0.67]; *P* < 0.001) (**Figure 4**). Neither the first-treatment-phase group (*P* = 0.79, I^2^= 0%) (MD= -1.01; 95%CI [-1.46, -0.55]; *P* < 0.001) nor the any-treatment-phase group (*P* = 0.19, I^2^= 41%) (MD= -1.79; 95%CI [-2.80, -0.79]; *P* < 0.001) showed significant heterogeneity, and the differences between the two groups were statistically significant (**Table 3**). Taken together, these exploratory observations suggested that effect sizes for psychological distress may vary across treatment stages; however, no formal test for subgroup differences was performed, and this interpretation remains hypothesis-generating only.

**Figure 4:**
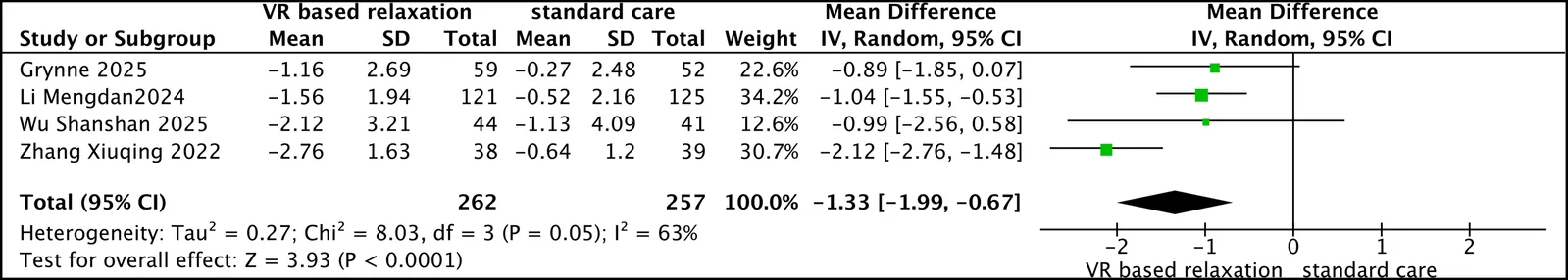
Meta-analysis of psychological distress levels among BCSs: VR-relaxation versus standard care.

### Risk of bias and sensitivity analyses

Publication bias was assessed for all included studies. The funnel plot suggested potential asymmetry; however, interpretation was limited by the small number of studies and the subjective nature of qualitative assessment. Therefore, quantitative tests were additionally performed. Across all three outcomes (anxiety, depression, and psychological distress), Egger’s tests did not identify statistically significant funnel plot asymmetry (all *P* > 0.05). Given the small number of studies contributing to each pooled estimate, both visual funnel plot inspection and Egger’s tests had limited statistical power to detect publication bias, and the possibility of underlying publication bias could not be fully excluded. The funnel plot and the results of Egger’s test are presented in the **Supplementary Materials**.

The sensitivity analysis results for anxiety and depression indicated no significant changes in effect size or heterogeneity. (**Table 4**) In terms of psychological distress, heterogeneity significantly decreased and became acceptable (*P* = 0.96, I²= 0%) after excluding one study (30). However, the number of studies included in each subgroup was limited, and the results of the sensitivity analysis were only provided for exploratory reference.

**Table 4:** Outcomes of the sensitivity analysis and test of publication bias.

Outcomes	No. of study	Sensitivity analysis		Egger’ s test
		SMDs (95% CI)	I^2^ (%)	*P* value
Anxiety	6	-1.94 (-2.49, -1.40) to -1.73 (-2.19, -1.26)	81 to 71	0.82
Depression	4	-1.11 (-1.36, -0.87) to -1.25 (-1.54, -0.96)	25 to 0	0.58
Psychological distress	4	-1.36 (-1.72, -1.00) to -1.01 (-1.44, -0.57)	63 to 0	0.55

### Qualitative analysis

A total of three studies (26, 30, 35) were included in the fatigue outcome. Nevertheless, given their significant heterogeneity (*P* < 0.01, I² = 87%), we only conducted a qualitative analysis of the results to minimize statistical errors. In the three studies regarding fatigue, the control group patients all received standard care. Zhang et al. (30) conducted VR relaxation training for breast cancer patients who had completed at least two chemotherapy cycles. The training was administered over a three-month period, with a minimum of three sessions per month during the initial phase. They found that, compared to conventional care, the VR group showed significantly greater improvement in fatigue. (t = -10.08, *P* < 0.001). Uslu et al. (26) exclusively enrolled breast cancer patients who were undergoing the CA chemotherapy regimen. The intervention involved viewing 360-degree natural beach scenery in a virtual environment. The training was administered over four cycles, with each cycle spanning 21 days. They found that participants in the second (t = -2.06, *P* = 0.044) and third cycles (t = −2.04, *P* = 0.045) experienced a reduction in fatigue (*P* < 0.05). Furthermore, improvements in physiological, emotional, and cognitive fatigue were observed relative to controls. Fu et al. (35) evaluated cancer-related fatigue among older breast cancer patients receiving CMF chemotherapy before and after VR intervention and reported significant fatigue reduction in the intervention group (t = -4.21, *P* < 0.001). Despite methodological differences, these three studies shared a core therapeutic strategy: VR scenarios to guide relaxation. All three trials showed greater fatigue improvements in VR groups versus controls.

### GRADE level

To preserve methodological rigor, we noted that fewer than six studies reported data for depression and psychological distress. With such a small number of studies, funnel plots and Egger’s tests had limited power to detect publication bias. We therefore downgraded the certainty of evidence for these two outcomes due to concerns about potential publication bias. Studies of anxiety showed substantial heterogeneity, corresponding to serious inconsistency. Furthermore, slight baseline imbalance was observed among trials assessing psychological distress, and this factor was considered during the risk-of-bias assessment. (**Supplementary Table 2**).

## Discussions

Consistent with the findings of Yazdipour (36), these results confirm that VR-ART suggest that VR-ART may help reduce anxiety in BCSs. Exploratory subgroup analyses revealed greater improvements in anxiety symptoms among patients in the initial treatment phase. Drawing on Kübler-Ross’s five-stage grief model, we hypothesize that patients in this phase predominantly fall within the bargaining or depression stage (37). Such patients tend to be more receptive to external psychological support, which may partly explain the stronger intervention effect observed here. Importantly, this study did not directly assess patients’ grief states; this theoretical interpretation remains speculative, derived only from our outcome findings and requires further validation in future research. A review of existing literature indicated that Zhang et al. (38) analysed 8 studies and confirmed that VR interventions produce beneficial impacts on anxiety symptom reduction and upper-extremity abduction recovery. Bani et al. (39) found that immersive VR distraction reduced pain and procedure-related anxiety in patients receiving standard analgesia, suggesting that it could serve as a complementary treatment.

Among BCSs, VR-ART was associated with statistically significant reductions in depressive symptoms. Supporting this finding, Zhang et al. (38) reported from their qualitative analysis that VR-based distraction interventions alleviated depressive symptoms among breast cancer patients, especially those with elevated baseline psychological distress. One included trial (34) demonstrated that VR intervention yielded greater antidepressant effects than music therapy, with benefits maintained throughout follow-up. Indovina et al. further noted that these therapeutic effects persisted for at least three treatment cycles (40). Bu et al. also analyzed 10 RCT studies and found that the application of VR significantly improved depression, anxiety, pain and upper limb function in patients with BCSs, although the heterogeneity of their results was relatively obvious (41). Compared with standard care, this combined approach addresses patients’ emotional recovery needs in several ways.

Qualitative synthesis indicated that VR relaxation therapy may alleviate fatigue among BCSs. The multisensory stimulation delivered within the virtual environment may support sustained beneficial effects. Previous work has identified chemotherapy cycles as an independent predictor of fatigue in BCSs (42). Drawing on this evidence alongside findings from sensitivity analyses, we speculated that chemotherapy regimens might partly contribute to variability in treatment effects for fatigue. Eid et al. (43) analysed 8 RCTs of patients with mixed-type cancers, showing VRER improved shoulder range-of-motion and lowered kinesiophobia versus conventional therapy. Two studies have confirmed that VR-ART can relieve sleep disorders in BCSs (27, 30). Future research should further validate the effectiveness of this therapy in addressing the entire symptom complex.

VR-ART was associated with reduced psychological distress among BCSs, with larger improvements observed in patients in later treatment stages relative to those in the initial treatment phase. This finding was also confirmed in the study by Saracino et al. (37) In the early treatment phase, psychological distress is often triggered by diagnostic shock and information overload, this heightened state of anxiety may weaken the benefits of VR-based distraction and relaxation. Several trials reported no significant between-group differences in psychological distress, potentially owing to short intervention and follow-up durations (32, 33). Heterogeneity decreased substantially after excluding one trial that used VR controllers (30). Based on this exploratory analysis, we speculated that VR interactivity might be associated with variability in treatment outcomes across studies. While our review and prior studies by Schneider and Persson reported low rates of VR-related sickness with this intervention (44, 45), other trials noted poorer adherence to VR interventions among older BCSs (32, 35). We hypothesized that this pattern might stem from limited device suitability and psychological apprehension among older adults. Because adverse events were not systematically documented in several included studies, we could not conclude that VR-ART was entirely free of risks.

## Advantages and limitations

Against prior meta-analyses on-VR rehabilitation, our analysis incorporated more studies per outcome indicator and achieved lower heterogeneity. We examined whether effect sizes differed across study-level characteristics, and these exploratory comparisons were hypothesis-generating only. Moreover, we restricted interventions exclusively to VR-ART by excluding VR-based general-health-education trials to strengthen outcome robustness. We also collected comprehensive negative-emotion indices and systematically synthesised intervention effects on emotional management.

Nevertheless, this review has limitations (1): Substantial between-study heterogeneity in reported effect sizes was observed, partially attributable to inherent variation in intervention design, delivery, and implementation across included trials. (2) This meta-analysis only included Chinese and English literature, which may introduce language bias. Moreover, it did not comprehensively cover grey literature, thus potentially introducing publication bias risk. (3) The subgroup analyses in this study were exploratory. As the number of studies included in each subgroup was less than 10, we did not perform a formal heterogeneity test, and the source of heterogeneity requires further validation.

## Conclusion

This systematic review and meta-analysis suggested that VR-ART may yield modest improvements in anxiety, depression and psychological distress among BCSs. Meanwhile, qualitative analysis indicated potential benefits of VR-ART for cancer-related fatigue. Key limitations included a small number of eligible RCTs, unexplored sources of between-study heterogeneity, and limited statistical power to assess publication bias for outcomes with few studies; accordingly, all pooled effect estimates were interpreted with caution. We adopted a broad survivorship definition covering patients receiving active treatment and those in post-treatment follow-up, and divergent symptom responses across treatment stages may contribute to some of the clinical heterogeneity. GRADE assessment rated the overall certainty of evidence as moderate. Although we reasonably speculated tentative associations between effect sizes and intervention timing, VR interactivity, and chemotherapy regimen, these findings were hypothesis-generating only. No factor could be confirmed as a definitive driver of treatment effects, and variations in patient experiences across relaxation modalities warranted further investigation.

Future investigations may continue to refine VR-ART for BCSs. Researchers could optimize VR content and relaxation modalities to identify regimens linked to improved symptom relief. Large multicenter standardized RCTs in clearly defined patient subgroups would be required to enhance evidence certainty. Longitudinal approaches may characterize psychological adaptation trajectories across the survivorship continuum to inform optimal intervention windows. Clinically, VR-ART may serve as a low-burden adjunct to conventional psychological care. Implementation research should explore individualized relaxation training dosing and develop age-friendly, accessible VR tools to boost engagement among older survivors, a group with historically low uptake of immersive interventions.

## Data Availability

The original contributions presented in the study are included in the article/**Supplementary Materials**. Further inquiries can be directed to the corresponding author.
