# Supplementary material for: The effectiveness of virtual reality–assisted relaxation therapy in improving psychological symptoms and fatigue of breast cancer survivors: a systematic review and meta-analysis
Source: Front Oncol. 2026 Oct 23;16:1927287. doi: 10.3389/fonc.2026.1927287 (PMC13739477; doi:10.3389/fonc.2026.1927287)
Supplement: Supplementary file 1 [file DataSheet1.docx]

**1.Search Strategy**

**English database**

PubMed

| #1 | ("Breast Neoplasms"[Mesh] OR "breast neoplasm*"[Title/Abstract] OR "breast tumor*"[Title/Abstract] OR "breast cancer*"[Title/Abstract] OR "mammary cancer*"[Title/Abstract] OR "Breast Malignant*"[Title/Abstract] OR "Mammary Carcinoma"[Title/Abstract] OR "Mammary Neoplasm"[Title/Abstract] OR "Breast Carcinoma"[Title/Abstract]) |
| --- | --- |
| #2 | ("Virtual Reality"[Mesh] OR "VR"[Title/Abstract] OR "Virtual Reality*" OR "Virtual Environment"[Title/Abstract] OR "Head-mounted Display"[Title/Abstract] OR "Digital Health"[Title/Abstract] OR "Virtual"[Title/Abstract] OR (VR[title/abstract] AND Psychological*[title/abstract]) OR (Virtual Reality*[title/abstract] AND Relaxation*[title/abstract])) |
| #3 | ("Emotions"[Mesh] OR "negative emotion"[Title/Abstract] OR "feeling*"[Title/Abstract] OR "Emotional adjustment"[Title/Abstract] OR "Psychological Rehabilitation"[Title/Abstract] OR Psychological[Title/Abstract] OR "anxiety" OR "depress*" OR "burden*" OR "stress*" OR fatigue OR fear OR "pain*" OR distress OR Happiness) |
| #4 | (Randomized Controlled Trials OR Clinical Trials OR Randomized Interventions OR RCT) |

Embase

| #1 | ('breast cancer'/exp OR 'breast cancer') AND [embase]/lim |
| --- | --- |
| #2 | ('virtual reality'/exp OR 'virtual reality') AND [embase]/lim |
| #3 | ('emotions'/exp OR 'emotions') AND [embase]/lim |
| #4 | 'breast neoplasm':ti,ab,kw OR 'breast tumor':ti,ab,kw OR 'breast cancer':ti,ab,kw OR 'mammary cancer':ti,ab,kw OR 'breast malignant':ti,ab,kw OR 'mammary carcinoma':ti,ab,kw OR 'mammary neoplasm':ti,ab,kw OR 'breast carcinoma':ti,ab,kw OR 'breast gland cancer or breast gland neoplasm or breast malignancies or breast malignancy or breast tumor malignant or ca breast or cancer in the mammary gland or cancer of the breast':ti,ab,kw |
| #5 | vr:ti,ab,kw OR 'virtual reality':ti,ab,kw OR 'virtual environment':ti,ab,kw OR 'head-mounted display':ti,ab,kw OR 'digital health':ti,ab,kw OR virtual:ti,ab,kw OR (vr:ti,ab,kw AND psychological:ti,ab,kw) OR ('virtual reality':ti,ab,kw AND relaxation:ti,ab,kw) |
| #6 | 'emotion or emotional factor or emotional response or emotional status or emotional structure':ti,ab,kw OR 'expressed emotion':ti,ab,kw OR 'negative emotion':ti,ab,kw OR feeling:ti,ab,kw OR 'emotional adjustment':ti,ab,kw OR 'psychological rehabilitation':ti,ab,kw OR anxiety:ti,ab,kw OR depress:ti,ab,kw OR burden:ti,ab,kw OR stress:ti,ab,kw OR fatigue:ti,ab,kw OR fear:ti,ab,kw OR pain:ti,ab,kw OR distress:ti,ab,kw OR happiness:ti,ab,kw |
| #7 | 'randomized controlled trials':ti,ab,kw OR 'clinical trials':ti,ab,kw OR 'randomized interventions':ti,ab,kw |
| #8 | #1 OR #4 |
| #9 | #2 OR #5 |
| #10 | #3 OR #6 |
| #11 | #7 AND #8 AND #9 AND #10 |

Cochrane

| #1 | Breast Neoplasms[Mesh] |
| --- | --- |
| #2 | breast neoplasm OR breast tumor OR breast cancer OR mammary cancer OR Breast Malignant OR Mammary Carcinoma OR Mammary Neoplasm OR Breast Carcinoma |
| #3 | #1 OR #2 |
| #4 | Virtual Reality[Mesh] |
| #5 | VR OR Virtual Reality OR Virtual Environment OR Head-mounted Display OR Digital Health OR Virtual OR (VR AND Psychological) OR (Virtual Reality AND Relaxation) |
| #6 | #4 OR #5 |
| #7 | Emotions[Mesh] |
| #8 | negative emotion OR feeling OR Emotional adjustment OR Psychological Rehabilitation OR anxiety OR depress OR burden OR stress OR fatigue OR fear OR pain OR distress OR Happiness |
| #9 | #7 OR #8 |
| #10 | Randomized Controlled Trials OR Clinical Trials OR Randomized Interventions |
| #11 | #3 AND #6 AND #9 AND #10 |

CINAL

| #1 | TI MH "Breast Neoplasms" OR TI (breast neoplasm OR breast tumor OR breast cancer OR mammary cancer OR Breast Malignant OR Mammary Carcinoma OR Mammary Neoplasm OR Breast Carcinoma) OR AB (breast neoplasm OR breast tumor OR breast cancer OR mammary cancer OR Breast Malignant OR Mammary Carcinoma OR Mammary Neoplasm OR Breast Carcinoma) |
| --- | --- |
| #2 | TI MH "Virtual Reality" OR TI (VR OR Virtual Reality OR Virtual Environment OR Head-mounted Display OR Digital Health OR Virtual OR (VR AND Psychological) OR (Virtual Reality AND Intervention)) OR AB (VR OR Virtual Reality OR Virtual Environment OR Head-mounted Display OR Digital Health OR Virtual OR (VR AND Psychological) OR (Virtual Reality AND Relaxation)) |
| #3 | TI MH "Emotions" OR TI (negative emotion OR feeling OR Emotional adjustment OR Psychological Rehabilitation OR anxiety OR depress OR burden OR stress OR fatigue OR fear OR pain OR distress OR Happiness) OR AB (negative emotion OR feeling OR Emotional adjustment OR Psychological Rehabilitation OR anxiety OR depress OR burden OR stress OR fatigue OR fear OR pain OR distress OR Happiness) |
| #4 | #1 AND #2 AND #3 |

WOS

| #1 | TS="Breast Neoplasms" OR "breast neoplasm*" OR "breast tumor*" OR "breast cancer*" OR "mammary cancer*" OR "Breast Malignant*" OR "Mammary Carcinoma" OR "Mammary Neoplasm" OR "Breast Carcinoma" |
| --- | --- |
| #2 | TS="Virtual Reality" OR "VR" OR "Virtual Reality*" OR "Virtual Environment" OR "Head-mounted Display" OR "Digital Health" OR "Virtual" OR (VR AND Psychological*) OR (Virtual Reality* AND relaxation*) |
| #3 | TS="Emotions" OR "negative emotion" OR "feeling*" OR "Emotional adjustment" OR "Psychological Rehabilitation" OR "anxiety" OR "depress*" OR "burden*" OR "stress*" OR "fatigue" OR "fear" OR "pain*"OR "distress" OR “Happiness” |
| #4 | TS= Randomized Controlled Trials OR Clinical Trials OR Randomized Interventions OR RCT |

**clinicaltrials.gov**

| **Search Query** | ("Breast Neoplasms" OR breast neoplasm OR breast tumor OR breast cancer OR mammary cancer OR "Breast Malignant" OR "Mammary Carcinoma" OR "Mammary Neoplasm" OR "Breast Carcinoma") AND ("Virtual Reality" OR VR OR "Virtual Environment" OR "Head-mounted Display" OR "Digital Health" OR Virtual OR (VR AND Psychological) OR ("Virtual Reality" AND Intervention) OR ("Virtual Reality" AND Relaxation)) AND ("Emotions" OR "negative emotion" OR feeling OR "Emotional adjustment" OR "Psychological Rehabilitation" OR anxiety OR depression OR burden OR stress OR fatigue OR fear OR pain OR distress OR Happiness) AND Randomized controlled trial |
| --- | --- |

**WHO ICTRP**

| **Search Query** | ("Breast Neoplasms" OR breast neoplasm OR breast tumor OR breast cancer OR mammary cancer OR "Breast Malignant" OR "Mammary Carcinoma" OR "Mammary Neoplasm" OR "Breast Carcinoma") AND ("Virtual Reality" OR VR OR "Virtual Environment" OR "Head-mounted Display" OR "Digital Health" OR Virtual OR (VR AND Psychological) OR ("Virtual Reality" AND Intervention) OR ("Virtual Reality" AND Relaxation)) AND ("Emotions" OR "negative emotion" OR feeling OR "Emotional adjustment" OR "Psychological Rehabilitation" OR anxiety OR depression OR burden OR stress OR fatigue OR fear OR pain OR distress OR Happiness) |
| --- | --- |

**GreyGuide**

| **Search Query** | "breast cancer" AND ("Virtual Reality" OR VR) AND (anxiety OR depress* OR stress OR distress OR fatigue) |
| --- | --- |

**Chinese database**

CNKI

| #1 | TKA=（‘乳腺肿瘤’+‘乳腺癌’+‘乳腺恶性肿瘤’） |
| --- | --- |
| #2 | TKA=（‘虚拟现实’+‘虚拟现实暴露疗法’+‘虚拟实景’+‘数字健康’+‘虚拟现实技术’+‘虚拟仿真’+‘虚拟环境’+‘头戴式显示器’+‘虚拟游戏’+‘分散注意力’+‘放松疗法’+‘交互式’+‘沉浸式’+‘VR技术’+‘虚拟情景’+‘虚拟’） |
| #3 | FT=（‘情绪’+‘情感障碍’+‘抑郁’+‘心理康复’+'心理治疗'+‘心理健康’+‘情绪调适’+‘心理干预’+‘心理护理’+‘心理问题’+‘不良情绪’+‘负性情绪’+‘焦虑’+‘疲乏’+‘恐惧’+‘痛苦’+‘紧张’+‘幸福感’） |

SinoMed

| #1 | (“乳腺肿瘤"[不加权:扩展] OR “乳腺肿瘤”[不加权:扩展] OR “乳腺癌” OR “乳腺恶性肿瘤”) |
| --- | --- |
| #2 | (“虚拟现实”[不加权:扩展] OR “虚拟现实”[不加权:扩展] OR “虚拟现实暴露疗法”[不加权:扩展] OR “虚拟实景” OR “数字健康” OR “虚拟现实技术” OR “虚拟仿真” OR “虚拟环境” OR “头戴式显示器” OR “虚拟游戏” OR “分散注意力” OR “放松疗法” OR “交互式模拟” OR “沉浸式模拟” OR “VR技术” OR “虚拟情景” OR “虚拟”) |
| #3 | (“情绪”[不加权:扩展] OR “情感障碍”[不加权:扩展] OR “抑郁”[不加权:扩展] OR “心理困扰”[不加权:扩展] OR “心理健康” OR “心理康复” OR “心理干预” OR “心理护理” OR “康复” OR “心理问题” OR “不良情绪” OR “负性情绪” OR “焦虑” OR “疲乏” OR “恐惧” OR “痛苦” OR “紧张” OR “幸福感”) |

Wanfang Data

| #1 | AB=（乳腺肿瘤 OR 乳腺癌 OR 乳腺恶性肿瘤） |
| --- | --- |
| #2 | AB=（虚拟现实 OR 虚拟现实暴露疗法 OR 虚拟实景 OR 数字健康 OR 虚拟现实技术 OR 虚拟仿真 OR 虚拟环境 OR 头戴式显示器 OR 虚拟游戏 OR 分散注意力 OR 放松疗法 OR 交互式 OR 沉浸式 OR VR技术 OR 虚拟情景 OR 虚拟） |
| #3 | AB=（情绪 OR 情感障碍 OR 抑郁 OR 心理困扰 OR 心理治疗 OR 心理康复 OR 心理健康 OR 情绪调适 OR 心理干预 OR 心理护理 OR 心理问题 OR 不良情绪 OR 负性情绪 OR 焦虑 OR 疲乏 OR 恐惧 OR 痛苦 OR 紧张 OR 幸福感) |
| #4 | AB=(随机对照试验OR 临床研究 OR 干预措施 OR 对照比较 ) |

VIP

| #1 | SU=（乳腺肿瘤 OR 乳腺癌 OR 乳腺恶性肿瘤） |
| --- | --- |
| #2 | SU=（虚拟现实 OR 虚拟现实暴露疗法 OR 虚拟实景 OR 数字健康 OR 虚拟现实技术 OR 虚拟仿真 OR 虚拟环境 OR 头戴式显示器 OR 虚拟游戏 OR 分散注意力 OR 放松疗法 OR 交互式 OR 沉浸式 OR VR技术 OR 虚拟情景 OR 虚拟） |
| #3 | SU=（情绪 OR 情感障碍 OR 抑郁 OR 心理困扰 OR 心理治疗 OR 心理康复 OR 心理健康 OR 情绪调适 OR 心理干预 OR 心理护理 OR 心理问题 OR 不良情绪 OR 负性情绪 OR 焦虑 OR 疲乏 OR 恐惧 OR 痛苦 OR 紧张 OR 幸福感） |

**2.Abstract translations of the 2 Chinese literatures included**

1*.***Title:** Study on the Application of PERMA Well-being Nursing Combined with Virtual Reality Technology in Rehabilitation Nursing after Breast Cancer Surgery

**Author and Publication Year**: Lizhen Yu 2024

**Journal of Publication and Website Address**: *Medical Innovation of China.21*(13), 83-87. http://dx.chinadoi.cn/10.3969/j.issn.1674-4985.2024.13.019.

**Abstract:**

**Objective**: To investigate the effect of PERMA well-being nursing combined with virtual reality (VR) technology in rehabilitation nursing after breast cancer surgery. **Method:** A total of 60 patients underwent breast cancer resection in Yingtan City People's Hospital from May 2020 to May 2023 were divided into two groups according to random number table method, with 30 cases in each group. Both groups were given 2 months of intervention. Patients in the control group received routine nursing, and the observation group received PERMA well-being nursing combined with VR technology intervention on the basis of the control group. The functional assessment of cancer therapy-breast cancer (FACT-B), self-rating anxiety scale (SAS), self-rating depression scale (SDS), general well-being schedule (GWB) and therapy adherence were compared between the two groups. **Result**: After intervention, FACT-B and GWB scores in observation group were higher than those in control group, SAS and SDS scores were lower than those in control group, the differences were statistically significant (P<0.05). The total therapy adherence of the observation group was significantly higher than that of the control group, the difference was statistically significant (P<0.05). **Conclusion**: PERMA well-being nursing combined with VR technology can obviously improve the quality of life and well-being of patients, improve their negative mental state and promote the rehabilitation of patients after breast cancer surgery.

2. **Title:** Effects of mindfulness intervention based on virtual reality technology on quality of life and cancer-related fatigue of elderly patients with breast cancer undergoing chemotherapy

**Author and Publication Year**: Sijia Fu, Linlin Qu 2025

**Journal of Publication and Website Address**: *Geriatrics Health Care* *31*(1), 42-46,52. http://dx.chinadoi.cn/10.3969/j.issn.1008-8296.2025.01.010.

**Abstract:**

**Objective**: To explore the effects of mindfulness intervention based on virtual reality technology on the quality of life and cancer-related fatigue (CRF) of elderly patients with breast cancer undergoing chemotherapy, and provide a method for treating this condition. **Methods:** A total of 122 elderly patients with breast cancer who received chemotherapy in The Affiliated Hospital of Northwest University, Xi'an No.3 Hospital from January 2021 to December 2023 were selected and divided into observation group(n=61) and control group(n=61) by random number table method. The control group received routine nursing, and the observation group received mindfulness intervention based on virtual reality technology on the basis of the control group. The quality of life [assessed by the Functional Assessment of Cancer Therapy-Breast (FACT-B) scale], CRF (assessed by the Cancer-Related Fatigue Scale) and immune function were compared between the two groups. **Results**: After intervention, the total scores and scores of each dimension of FACT-B scale, as well as the levels of CD3+ and CD4+ in both groups were higher than those in the same group before intervention, and the observation group was higher than the control group, with statistical significance(P＜0.05); CRF score and CD8+ level were lower than those in the same group before intervention, and the observation group was lower than the control group, with statistically significant differences(P＜ 0.05). **Conclusion**: The mindfulness intervention based on virtual reality technology can effectively improve the quality of life and immune function of elderly patients with breast cancer undergoing chemotherapy and alleviate their CRF. It has certain clinical application value.

**Supplementary Fig. 1 Risk of Bias of The Included Studies (ROB2)**

**Supplementary TABLE 1 The original data and the transformed change values of emotional indicators in each study**

| **anxiety** | | | | | | | | | |
| --- | --- | --- | --- | --- | --- | --- | --- | --- | --- |
| transformed change values | | | | | | | original data | | |
| study | emean | esd | en | cmean | csd | cn | Baseline (VR/CG) | Destination (VR-ART) | Destination (CG) |
| Chirico 2020 | -6.86 | 1.75 | 28 | -1.47 | 1.56 | 34 | 44.296(1.808)/45.412(1.611) | 37.444(1.684) | 43.941(1.50) |
| Czech 2024 | -5.88 | 3.96 | 17 | 0.69 | 4.21 | 16 | 9.29(4.48)/7.06(4.81) | 3.41 (3.02) | 7.75 (1.77) |
| Torres 2024 | -11.66 | 2.19 | 71 | -7 | 1.37 | 62 | 17.63(0.68)/18.64(0.69) 18 (16–19)/19 (17–20) | 5.96(0.45) 6 (5–7) | 11.63(0.69) 12 (10–13) |
| Uslu 2023 | -14.81 | 10.04 | 33 | 2.67 | 11.14 | 33 | 42.45(11.58)/41.15(9.18) | 27.64(5.30) | 43.82(12.39) |
| Yu Lizhen 2024 | -10.33 | 5.3 | 30 | -3.99 | 3.95 | 30 | 56.62(3.85)/55.28(4.13) | 46.29(3.64) | 51.29(3.74) |
| Zhang Xiuqing 2022 | -7.5 | 11.17 | 38 | 5.9 | 5.91 | 39 | 51.66(11.252)/49.31(6.814) | 44.16(11.083) | 55.21(3.806) |
| **depression** | | | | | | | | | |
| transformed change values | | | | | | | original data | | |
| study | emean | esd | en | cmean | csd | cn | Baseline (VR/CG) | Destination (VR-ART) | Destination (CG) |
| Czech 2024 | -3.06 | 3.35 | 17 | 3.19 | 4.27 | 16 | 5.59(3.83 )/4.81(4.92) | 2.53 (2.40) | 8.00 (2.19) |
| Torres 2024 | -8.61 | 1.93 | 71 | -6.45 | 1.58 | 62 | 16.94(0.91)/19.31(0.71) 17 (15–19)/19 (18–21) | 8.16(1.14)8 (6–11) | 12.63(0.69) 13 (11–14) |
| Yu Lizhen 2024 | -9.74 | 3.87 | 30 | -4.98 | 4.11 | 30 | 54.21(4.07)/53.67(4.38) | 44.47(3.64) | 48.69(3.76) |
| Zhang Xiuqing 2022 | -4.69 | 10.79 | 38 | 1.57 | 4.47 | 39 | 51.32(11.552)/48.64(4.934) | 46.63(9.824) | 50.21(3.806) |
| **fatigue** | | | | | | | | | |
| transformed change values | | | | | | | original data | | |
| study | emean | esd | en | cmean | csd | cn | Baseline (VR/CG) | Destination (VR-ART) | Destination (CG) |
| Fu Sijia 2025 | -15.14 | 9.67 | 61 | -9.38 | 4.56 | 61 | 37.06(9.46)/37.67(9.41) | 21.92(6.22) | 28.29(7.92) |
| Uslu 2023 | -2.94 | 8.62 | 33 | 3.67 | 9.87 | 33 | 18.27(9.38)/15.94(7.42) | 15.33(7.56) | 19.61(11.20) |
| Zhang Xiuqing 2022 | -25.24 | 14.43 | 38 | -1.85 | 8.41 | 39 | 103.03(8.645)/101.62(9.35) | 77.79(11.548) | 99.77(6.964) |
| **psychological distress** | | | | | | | | | |
| transformed change values | | | | | | | original data | | |
| study | emean | esd | en | cmean | csd | cn | Baseline (VR/CG) | Destination (VR-ART) | Destination (CG) |
| Grynne 2025 | -1.16 | 2.69 | 59 | -0.27 | 2.48 | 52 | 3.72(2.88)/3.25(2.50) | 2.56(2.44) | 2.98(2.45) |
| Li Mengdan 2024 | -1.56 | 1.94 | 121 | -0.52 | 2.16 | 125 | 4.83 (2.05)/5.15 (2.35) | 3.27 (1.81) | 4.63 (1.91) |
| Wu Shanshan 2025 | -2.12 | 3.21 | 44 | -1.13 | 4.09 | 41 | 3.961(3.17)/3.39(4.64) 3.0 (2.0, 5.0)/2.0 (1.0, 4.0) | 1.84(3.25) 1.0 (0.3, 2.0) | 2.26(3.10) 2.0 (0, 2.5) |
| Zhang Xiuqing 2022 | -2.76 | 1.63 | 38 | -0.64 | 1.2 | 39 | 5.71(1.250)/6.13(0.695) | 2.95(1.845) | 5.49(1.393) |
| The data represented as median, quartile and range in the literature have been marked in red. | | | | | | | |  |  |

Some of the original studies only provided the baseline Mean(Mean(B)), baseline SD(SD(B)), final Mean(Mean(F)), and final SD(SD(F)). To convert these values, the following formulas need to be used, thereby obtaining the difference Mean and difference SD(r=0.5).

Mean=Mean(B)$-$Mean(F), SD=$\sqrt{{SD\left( B \right)}^{2}+{SD\left( F \right)}^{2}-(2\times R\times SD\left( B \right)\times SD\left( F \right))}$.

Standard deviations were estimated from reported IQRs as SD = (Q3 − Q1)/1.35, and from reported total ranges as SD = (maximum − minimum)/4.

**Author(s):** Yu Deng and Jiangling Mo

**Question:** The Effectiveness of Virtual Reality Combined with Relaxation Therapy in Improving Negative Emotions of Breast Cancer Survivors

**Setting:** Virtual Reality Combined with Relaxation Therapy

**Bibliography:**

**Supplementary TABLE 2** Grade Checklist

| **Certainty assessment** | | | | | | | **№ of patients** | | **Effect** | | **Certainty** | **Importance** |
| --- | --- | --- | --- | --- | --- | --- | --- | --- | --- | --- | --- | --- |
| **№ of studies** | **Study design** | **Risk of bias** | **Inconsistency** | **Indirectness** | **Imprecision** | **Other considerations** | **VR-relaxation** | **Standard Care** | **Relative (95% CI)** | **Absolute (95% CI)** |  |  |
| **Anxiety** | | | | | | | | | | | | |
| 6 | randomised trials | not serious | serious ^a^ | not serious | not serious | none | 217 | 214 | - | SMD **1.94 SD lower** (2.49 lower to 1.4 lower) | ⨁⨁⨁◯ Moderate ^a^ | CRITICAL |
| **Depression** | | | | | | | | | | | | |
| 4 | randomised trials | serious ^b^ | not serious | not serious | not serious | none | 156 | 147 | - | SMD **1.11 SD lower** (1.36 lower to 0.87 lower) | ⨁⨁⨁◯ Moderate ^b^ | CRITICAL |
| **Psychological Distress** | | | | | | | | | | | | |
| 4 | randomised trials | serious ^d^ | not serious ^e^ | not serious | not serious | none | 262 | 257 | - | MD **1.36 SD lower** (1.72 lower to 1 lower) | ⨁⨁⨁◯ Moderate ^d,e^ | CRITICAL |

**GRADE assessment was not applicable for fatigue, as GRADE requires pooled effect estimates; CI:** confidence interval; **MD:** mean difference; **SMD:** standardised mean difference

#### Explanations

a. I2 =81%, p<0.0001

b. The number of included studies was relatively small.

c. I2 =87%, p<0.01

d. The baseline differences between the two studies were significant.

e. I2 =63%, p=0.05 After subgroup analysis, the heterogeneity was acceptable.

**Quantitative and Qualitative Tests for the Publication Bias of Anxiety**

Number of studies = 6	Root MSE	=	2.54

------------------------------------------------------------------------------

Std_Eff | Coefficient Std. err. t	P>|t|	[95% conf. interval]

-------------+----------------------------------------------------------------

slope | -1.566555	1.577349	-0.99	0.377	-5.945977	2.812867

bias | -1.304107	5.398026	-0.24	0.821	-16.29143	13.68321

------------------------------------------------------------------------------

Test of H0: no small-study effects	P = 0.821

**Quantitative and Qualitative Tests for the Publication Bias of Depression**

Number of studies = 4	Root MSE	=	1.319

------------------------------------------------------------------------------

Std_Eff | Coefficient Std. err. t	P>|t|	[95% conf. interval]

-------------+----------------------------------------------------------------

slope | -.7137074	.6446089	-1.11	0.384	-3.487236	2.059821

bias | -1.708348	2.589496	-0.66	0.577	-12.85005	9.433355

------------------------------------------------------------------------------

Test of H0: no small-study effects	P = 0.577

**Quantitative and Qualitative Tests for the Publication Bias of Psychological Distress**

Number of studies = 4	Root MSE	=	2.53

------------------------------------------------------------------------------

Std_Eff | Coefficient Std. err. t	P>|t|	[95% conf. interval]

-------------+----------------------------------------------------------------

slope |	.0486439	.8736272	0.06	0.961	-3.71027	3.807558

bias | -3.431733	4.841087	-0.71	0.552	-24.26125	17.39778

------------------------------------------------------------------------------

Test of H0: no small-study effects	P = 0.552

**A**

**B**

**Supplementary Fig. 2 Meta-Analysis of Anxiety Levels Among Breast Cancer Survivors:** (A) Any Treatment Phase; (B) First Treatment Phase

**C**

**D**

**Supplementary Fig. 3 Meta-Analysis of Psychological Distress Levels Among Breast Cancer Survivors:** (C) Any Treatment Phase; (D) First Treatment Phase

**Supplementary TABLE 3** PRISMA 2020 Checklist

| **Section and Topic** | **Item #** | **Checklist item** | **Location where item is reported** |
| --- | --- | --- | --- |
| **TITLE** | | |  |
| Title | 1 | Identify the report as a systematic review. | P1 |
| **ABSTRACT** | | |  |
| Abstract | 2 | See the PRISMA 2020 for Abstracts checklist. | P1-2 |
| **INTRODUCTION** | | |  |
| Rationale | 3 | Describe the rationale for the review in the context of existing knowledge. | P2-4 |
| Objectives | 4 | Provide an explicit statement of the objective(s) or question(s) the review addresses. | P4 |
| **METHODS** | | |  |
| Eligibility criteria | 5 | Specify the inclusion and exclusion criteria for the review and how studies were grouped for the syntheses. | P6 |
| Information sources | 6 | Specify all databases, registers, websites, organisations, reference lists and other sources searched or consulted to identify studies. Specify the date when each source was last searched or consulted. | P5 |
| Search strategy | 7 | Present the full search strategies for all databases, registers and websites, including any filters and limits used. | Supplementary Materials |
| Selection process | 8 | Specify the methods used to decide whether a study met the inclusion criteria of the review, including how many reviewers screened each record and each report retrieved, whether they worked independently, and if applicable, details of automation tools used in the process. | P5,P8 |
| Data collection process | 9 | Specify the methods used to collect data from reports, including how many reviewers collected data from each report, whether they worked independently, any processes for obtaining or confirming data from study investigators, and if applicable, details of automation tools used in the process. | P6-7 |
| Data items | 10a | List and define all outcomes for which data were sought. Specify whether all results that were compatible with each outcome domain in each study were sought (e.g. for all measures, time points, analyses), and if not, the methods used to decide which results to collect. | P6-7 |
|  | 10b | List and define all other variables for which data were sought (e.g. participant and intervention characteristics, funding sources). Describe any assumptions made about any missing or unclear information. | P7 |
| Study risk of bias assessment | 11 | Specify the methods used to assess risk of bias in the included studies, including details of the tool(s) used, how many reviewers assessed each study and whether they worked independently, and if applicable, details of automation tools used in the process. | P7 |
| Effect measures | 12 | Specify for each outcome the effect measure(s) (e.g. risk ratio, mean difference) used in the synthesis or presentation of results. | P6-7 |
| Synthesis methods | 13a | Describe the processes used to decide which studies were eligible for each synthesis (e.g. tabulating the study intervention characteristics and comparing against the planned groups for each synthesis (item #5)). | N |
|  | 13b | Describe any methods required to prepare the data for presentation or synthesis, such as handling of missing summary statistics, or data conversions. | P7 |
|  | 13c | Describe any methods used to tabulate or visually display results of individual studies and syntheses. | P8 |
|  | 13d | Describe any methods used to synthesize results and provide a rationale for the choice(s). If meta-analysis was performed, describe the model(s), method(s) to identify the presence and extent of statistical heterogeneity, and software package(s) used. | P8 |
|  | 13e | Describe any methods used to explore possible causes of heterogeneity among study results (e.g. subgroup analysis, meta-regression). | P8 |
|  | 13f | Describe any sensitivity analyses conducted to assess robustness of the synthesized results. | P8 |
| Reporting bias assessment | 14 | Describe any methods used to assess risk of bias due to missing results in a synthesis (arising from reporting biases). | N |
| Certainty assessment | 15 | Describe any methods used to assess certainty (or confidence) in the body of evidence for an outcome. | P7 |

| **Section and Topic** | **Item #** | **Checklist item** | **Location where item is reported** |
| --- | --- | --- | --- |
| **RESULTS** | |  |  |
| Study selection | 16a | Describe the results of the search and selection process, from the number of records identified in the search to the number of studies included in the review, ideally using a flow diagram. | Figure1 |
|  | 16b | Cite studies that might appear to meet the inclusion criteria, but which were excluded, and explain why they were excluded. | P8 |
| Study characteristics | 17 | Cite each included study and present its characteristics. | Table1 |
| Risk of bias in studies | 18 | Present assessments of risk of bias for each included study. | Figure2 |
| Results of individual studies | 19 | For all outcomes, present, for each study: (a) summary statistics for each group (where appropriate) and (b) an effect estimate and its precision (e.g. confidence/credible interval), ideally using structured tables or plots. | Supplementary Materials |
| Results of syntheses | 20a | For each synthesis, briefly summarise the characteristics and risk of bias among contributing studies. | P12-15 |
|  | 20b | Present results of all statistical syntheses conducted. If meta-analysis was done, present for each the summary estimate and its precision (e.g.  confidence/credible interval) and measures of statistical heterogeneity. If comparing groups, describe the direction of the effect. | P12-15 |
|  | 20c | Present results of all investigations of possible causes of heterogeneity among study results. | P12-15 |
|  | 20d | Present results of all sensitivity analyses conducted to assess the robustness of the synthesized results. | P17 |
| Reporting biases | 21 | Present assessments of risk of bias due to missing results (arising from reporting biases) for each synthesis assessed. | N |
| Certainty of evidence | 22 | Present assessments of certainty (or confidence) in the body of evidence for each outcome assessed. | P19-20 |
| **DISCUSSION** | |  |  |
| Discussion | 23a | Provide a general interpretation of the results in the context of other evidence. | P17-19 |
|  | 23b | Discuss any limitations of the evidence included in the review. | P20 |
|  | 23c | Discuss any limitations of the review processes used. | P20 |
|  | 23d | Discuss implications of the results for practice, policy, and future research. | P20-21 |
| **OTHER INFORMATION** | |  |  |
| Registration and protocol | 24a | Provide registration information for the review, including register name and registration number, or state that the review was not registered. | P4 |
|  | 24b | Indicate where the review protocol can be accessed, or state that a protocol was not prepared. | P4 |
|  | 24c | Describe and explain any amendments to information provided at registration or in the protocol. | P4 |
| Support | 25 | Describe sources of financial or non-financial support for the review, and the role of the funders or sponsors in the review. | P21 |
| Competing  interests | 26 | Declare any competing interests of review authors. | P21 |
| Availability of data, code and other materials | 27 | Report which of the following are publicly available and where they can be found: template data collection forms; data extracted from included studies; data used for all analyses; analytic code; any other materials used in the review. | N |

*From:* Page MJ, McKenzie JE, Bossuyt PM, Boutron I, Hoffmann TC, Mulrow CD, et al. The PRISMA 2020 statement: an updated guideline for reporting systematic reviews. BMJ 2021;372:n71. doi: 10.1136/bmj.n71

For more information, visit: <http://www.prisma-statement.org/>

| **Supplementary TABLE 4 Characteristics of The Included Studies** | | | | | | | | | | | | |
| --- | --- | --- | --- | --- | --- | --- | --- | --- | --- | --- | --- | --- |
| Study | Area | Stage | n, M/F | Accepted Treatment | Treatment Time | Follow-up | Groups | Case | Age | Main Outcomes | Types of relaxation | Intervention |
| Uslu 2023 | Turkey | Any | 66, 0/66 | CA protocol in chemotherapy | 4 cycles | NR | VR | 33 | 53.42（9.25） | STAI, CFS | Natural environment relaxation training | Wear VR glasses to watch panoramic beach and nature content collected from the YouTube library, listen to natural sounds to relax, for 30 minutes each time. |
|  |  |  |  |  |  |  | SC | 33 | 51.42（8.53） |  |  |  |
| Czech 2024 | Poland | Any | 33, 0/33 | Any | 2 weeks | 8 weeks | VR | 17 | 56.68 (11.26) | HADS-M | Natural environment relaxation training | Receive virtual therapy garden training (VR TierOne) through a VR device, accompanied by relaxation training through visual, auditory and kinesthetic means, for a total of 8 sessions, each lasting 15 minutes. |
|  |  |  |  |  |  |  | SC | 16 | 59.88 (10.72) |  |  |  |
| Li Mengdan 2024 | China | First Chemotherapy | 246, 0/246 | Chemotherapy | 3 months | 6 months | VR | 121 | 53.6 (9.4) | DT | Natural environment relaxation training and information guidance | Using VR devices and headphones, three selectable virtual environments are created: forest, beach, and sea. With voice guidance, relaxation is facilitated. Once or twice a week, for 15 to 20 minutes each time. At least 12 interventions should be received before T1. |
|  |  |  |  |  |  |  | SC | 125 | 55.2 (9.7) |  |  |  |
| Torres 2024 | Spain | First Chemotherapy | 133, 0/133 | Chemotherapy | 4 cycles | NR | VR | 71 | 49 (11.7） | HADS-M | Natural environment relaxation training and information guidance | Using VR equipment, three relaxation training modules are provided, including: underwater environment, simulated chemotherapy scenario, and seasonal scenes. There are 4 repetitions of intervention, each lasting 30 to 45 minutes. |
|  |  |  |  |  |  |  | SC | 62 | 50 (11.6） |  |  |  |
| **Chemotherapy acronyms:** **CA**, cyclophosphamide plus doxorubicin; **CMF**, cyclophosphamide-methotrexate-flourouracil; **Other acronyms:** **F**, female; **M**, male; **NR**, not reported; **STAI**, State-Trait Anxiety Inventory scores; **CFS**, Cancer Fatigue Scale; **PFS**, Piper Fatigue Scale; **HADS-M**, Hospital Anxiety and Depression Scale; **DT**, Distress Thermometer; **SAS**, Self-Rating Anxiety Scale; **SDS**, Self-rating Depression Scale; **SAI**, Spielberger State Anxiety Inventory. | | | | | | | | | | | | |
| **Supplementary TABLE 4 (Continued)** | | | | | | | | | | | | |
| Study | Area | Stage | n, M/F | Accepted Treatment | Treatment Time | Follow-up | Groups | Case | Age | Main Outcomes | Types of relaxation | Intervention |
| Zhang Xiuqing 2022 | China | Any | 77, 0/77 | ≥2 courses of Chemotherapy | 3 months | NR | VR | 38 | 52.29（7.69） | DT, PFS, SAS, SDS | Natural environment relaxation training and information guidance | Using VR equipment to provide immersive environments such as the seaside and the butterfly valley, allowing walking, and enabling the hearing of wind sounds and intermittent guidance, touching butterflies, for 6 times, 30 minutes each time. At least 3 times should be received in the first month. |
|  |  |  |  |  |  |  | SC | 39 | 51.03 （7.98） |  |  |  |
| Yu Lizhen 2024 | China | Any | 60, 0/60 | First unilateral mastectomy | 2 months | NR | VR | 30 | 47.29（3.67 | SAS, SDS | Natural environment relaxation training and mindfulness techniques | Nurse conducted PERMA happiness care conversations with the patient, while using VR technology to create natural scenes such as forests, beaches, and wilderness. Interactive elements were added to the scenes. There were a total of 7 sessions, each lasting 30 minutes, once a week. |
|  |  |  |  |  |  |  | SC | 30 | 46.14（4.09） |  |  |  |
| Wu Shanshan 2025 | China | Any | 85, 0/85 | Chemotherapy | ( 5 ± 2 ) weeks | NR | VR | 44 | 43.6 (11.1) | DT | Natural environment relaxation training | Using VR equipment to provide natural landscapes combined with music and relaxation guidance, the scenes include natural environments such as trees, flowers, mountains and sunsets, for a total of 3 to 5 times, with each session lasting (15 ± 3) minutes. |
|  |  |  |  |  |  |  | SC | 41 | 49.3 (9.4) |  |  |  |
| **Chemotherapy acronyms:** **CA**, cyclophosphamide plus doxorubicin; **CMF**, cyclophosphamide-methotrexate-flourouracil; **Other acronyms:** **F**, female; **M**, male; **NR**, not reported; **STAI**, State-Trait Anxiety Inventory scores; **CFS**, Cancer Fatigue Scale; **PFS**, Piper Fatigue Scale; **HADS-M**, Hospital Anxiety and Depression Scale; **DT**, Distress Thermometer; **SAS**, Self-Rating Anxiety Scale; **SDS**, Self-rating Depression Scale; **SAI**, Spielberger State Anxiety Inventory. | | | | | | | | | | | | |
| **Supplementary TABLE 4 (Continued)** | | | | | | | | | | | | |
| Study | Area | Stage | n, M/F | Accepted Treatment | Treatment Time | Follow-up | Groups | Case | Age | Main Outcomes | Types of relaxation | Intervention |
| Grynne 2025 | Sweden | First Radiotherapy | 111, 0/111 | Radiotherapy | 2 weeks | 6 months | VR | 59 | 60.2（10.7） | DT | VR exposure therapy and cognitive relaxation | Using VR devices to provide simulated radiotherapy environments, and offering relaxation guidance and health information through animations, cards, and other means. Provided before and after each radiotherapy session, for 15 to 20 minutes per session. |
|  |  |  |  |  |  |  | SC | 52 | 61.7（9.18） |  |  |  |
| Chirico 2020 | Italy | First Chemotherapy | 62, 0/62 | CA protocol in adjuvant chemotherapy | 4 cycles | NR | VR | 28 | 55.18（5.70） | SAI | Natural environment relaxation training | The virtual environment includes: exploring an island, passing through the forest, observing animals, climbing mountains and swimming in the sea. It is conducted once every cycle, lasting for 20 minutes each time. |
|  |  |  |  |  |  |  | SC | 34 | 56.2（6.79） |  |  |  |
| Fu Sijia 2025 | China | Any | 122, 0/122 | CMF protocol in chemotherapy | 8 weeks | NR | VR | 61 | 72.5（6.20） | CFS | Natural environment relaxation training and mindfulness techniques | Before and after chemotherapy, use VR equipment to perform the following activities: 1. Sit in meditation: the scene is "Silent Forest"; 2. Body scan: the scene is "Zen Meditation Room"; 3. Mindfulness yoga: the scene is "Yoga Room"; 4. Mindfulness relaxation: the scene is "Forest"; 5. Mindfulness walking: the scene is "Deep Mountain Temple"; 6. Immersive stress reduction: the scene is "Beautiful Scenery". 1-2 times per day, 25 minutes per session. |
|  |  |  |  |  |  |  | SC | 61 | 72.9（6.10） |  |  |  |
| **Chemotherapy acronyms:** **CA**, cyclophosphamide plus doxorubicin; **CMF**, cyclophosphamide-methotrexate-flourouracil; **Other acronyms:** **F**, female; **M**, male; **NR**, not reported; **STAI**, State-Trait Anxiety Inventory scores; **CFS**, Cancer Fatigue Scale; **PFS**, Piper Fatigue Scale; **HADS-M**, Hospital Anxiety and Depression Scale; **DT**, Distress Thermometer; **SAS**, Self-Rating Anxiety Scale; **SDS**, Self-rating Depression Scale; **SAI**, Spielberger State Anxiety Inventory. | | | | | | | | | | | | |
